# Sublethal Effects of Triflumezopyrim on Biological Traits and Detoxification Enzyme Activities in the Small Brown Planthopper *Laodelphax striatellus* (Hemiptera: Delphacidae)

**DOI:** 10.3389/fphys.2020.00261

**Published:** 2020-04-07

**Authors:** Shuirong Zhang, Xuegui Wang, Fuchuan Gu, Changwei Gong, Lin Chen, Yuming Zhang, Ali Hasnain, Litao Shen, Chunxian Jiang

**Affiliations:** ^1^National Demonstration Center for Experimental Crop Science Education, Sichuan Agricultural University, Chengdu, China; ^2^Biorational Pesticide Research Laboratory, Sichuan Agricultural University, Chengdu, China

**Keywords:** *Laodelphax striatellus*, triflumezopyrim, sublethal effect, detoxification enzyme activity, vitellogenin

## Abstract

The small brown planthopper [*Laodelphax striatellus* (Fallén) (Hemiptera, Delphacidae)] is one of the most destructive insect pests of rice and has developed strong resistance to several kinds of chemical insecticides. Triflumezopyrim, a novel mesoionic insecticide developed by Corteva Agriscience (formerly DuPont Crop Protection), has efficient biological activity in controlling sucking insects, such as the planthopper. However, the effects of triflumezopyrim on the growth and reproduction of *L. striatellus* have not been reported. In this study, an F_5_ generation was obtained by conducting five rounds of insecticide screening on a sensitive *L. striatellus* strain (F_0_ generation). An age-stage life table procedure was used to evaluate the effects of a sublethal concentration (LC_50_) of triflumezopyrim on the biological parameters of *L. striatellus*. Compared with those of the F_0_ generation, the intrinsic rate of increase (*r*), the finite rate (λ), and the net reproductive rate (*R*_0_) of the F_5_ generation were significantly decreased; nevertheless, the average duration of life (*T*) was not significantly affected. The results of detoxification enzyme activity assays indicated that the glutathione *S*-transferase and cytochrome P450 monooxygenase (P450) activities in the F_5_ generation were significantly higher than those in the F_0_ generation. The contents of vitellogenin (Vg) and vitellogenin receptor (VgR) were also detected, and the results indicated that the contents of Vg and VgR in the F_5_ generation were significantly decreased compared to those in the F_0_ generation. Furthermore, we detected the relative expression of ecdysone receptor (EcR), Vg, and VgR in the F_0_ and F_5_ generations and found that the relative expression levels of Vg and VgR in the F_5_ generation female adults were obviously lower than those in the F_0_ generation (*P* < 0.05), whereas the relative expression of EcR was slightly increased, although the difference was not significant (*P* > 0.05). Based on these results, a sublethal concentration (median lethal concentration, LC_50_) of triflumezopyrim may inhibit the generational growth and reproduction of *L. striatellus*. Moreover, our results may provide a reference for further studies of the suitability and resistance mechanisms of *L. striatellus* subjected to a sublethal dose of triflumezopyrim.

## Introduction

The small brown planthopper [*Laodelphax striatellus* (Fallén) (Hemiptera: Delphacidae)] is one of the most destructive insect pests of rice; its adults and nymphs not only feed directly on rice but also spread viral diseases, such as rice black-streaked dwarf disease (Kisimoto, 1967; Yin et al., 2013). At present, the control of *L. striatellus* in China depends mainly on chemical insecticides, the use of which has resulted in different levels of resistance to many pesticides, such as deltamethrin, chlorpyrifos, imidacloprid, and buprofezin (Zhang et al., 2016; Abdalla Elzaki et al., 2018; Asaduzzaman et al., 2019). Triflumezopyrim is a newly commercialized molecule from Corteva Agriscience. The biochemical and physiological action of this novel insecticide involves its binding to the orthosteric site of the nicotinic acetylcholine receptor by competitive binding, making the insects lethargic and poisoned, which was highly effective for controlling both imidacloprid-susceptible and imidacloprid-resistant planthopper populations in Malaysia (Cordova et al., 2016). For Chinese rice planthopper populations, triflumezopyrim has extremely high efficacy; additionally, triflumezopyrim is less harmful to natural enemies, such as *Anagrus nilaparvatae* (Hymenoptera: Mymaridae), *Cyrtorhinus lividipennis* (Hemiptera: Miridae), and *Paederus fuscipes* (Coleoptera: Staphylinidae) (Zhu et al., 2018).

The occurrence of resistance is closely related to the sublethal effects of pesticides on insects and the changes in detoxification enzymes in insects (Ma et al., 2019; Meng et al., 2019a, b). The study of sublethal effects can be carried out by establishing an insect’s amphoteric life table. Sublethal effects are manifested mainly in the growth and development of insect individuals and their offspring, feeding behavior, mating and reproduction, oviposition, egg hatching rate, and population growth (Ramirez-Romero et al., 2007). Vitellin (Vn) is a specific protein of the female hemolymph that provides nutritional sources for embryonic development and is closely related to female insect reproduction (Sappington and Raikhel, 1998; Tufail and Takeda, 2008; Veerana et al., 2015; Liao et al., 2019; Xiang et al., 2019; Xu et al., 2019). Vitellogenin (Vg) is the necessary precursor material for synthesizing Vn. When developing oocytes take up Vg, they require membrane-bound Vg receptor (VgR) for transport (Schneider, 1996; Snigirevskaya et al., 1997). Ecdysone receptor (EcR) can inhibit the expression of Vg (Yan et al., 2016; Shen et al., 2018). Hence, the expression of the EcR, Vg, and VgR genes has a significant influence on insect growth and reproduction.

Our objective is to analyze the population dynamics, detoxification enzyme activities, Vg and VgR contents, and relative expression of the EcR, Vg, and VgR genes of *L. striatellus* under sublethal concentrations of triflumezopyrim. The findings of this study may provide a reference for further research on the suitability and resistance mechanisms of *L. striatellus* subjected to a sublethal dose of triflumezopyrim and provide theoretical support for the popularization and application of new pesticides.

## Materials and Methods

### Insects and Insecticide

A susceptible laboratory strain of *L. striatellus* (F_0_) was kindly provided by Nanjing Agricultural University in September 2016. These insects had not been exposed to any insecticides for 15 consecutive years and were continuously reared on rice seedlings (TN1) in our laboratory under a temperature of 27 ± 1°C, relative humidity of 70 ± 10%, and photoperiod of 14 L:10 D. Triflumezopyrim SC (10%) was purchased from Corteva Agriscience (Wilmington, DE, United States). All other chemicals and solvents used were analytical reagents.

### Bioassays

The toxicity of triflumezopyrim to *L. striatellus* was assayed using the rice seedling dipping method, with minor modifications (Xiang et al., 2019). A series of concentrations of triflumezopyrim were diluted with water containing 0.1% Triton^TM^ X-100, and 0.1% Triton X-100 solution was used as the control. After soaking the rice seedlings in the prepared liquid for 30 s, they were placed in newspaper to dry. The roots were wrapped with degreased cotton, sufficient water was absorbed, and they were placed in plastic cups. Fifteen healthy and uniform 3rd-instar nymphs were prepared for each treatment, and each concentration was tested in triplicate. After 96 h, the mortality rate of the test insects was counted (Liao et al., 2017), with direct death or incompatible movement as death. The toxicity regression equation *Y* = *a* + *bX*, standard error, LC_50_ value, 95% confidence interval (95% CI), χ^2^ value, and *Pr* were calculated using SAS 9.2 (North Carolina State University, Raleigh, North Carolina, United States).

### Sublethal Effects of Triflumezopyrim on *L. striatellus*

#### Triflumezopyrim Effects on the Life-History Traits of the F_4_ Generation

**FIGURE 1:**
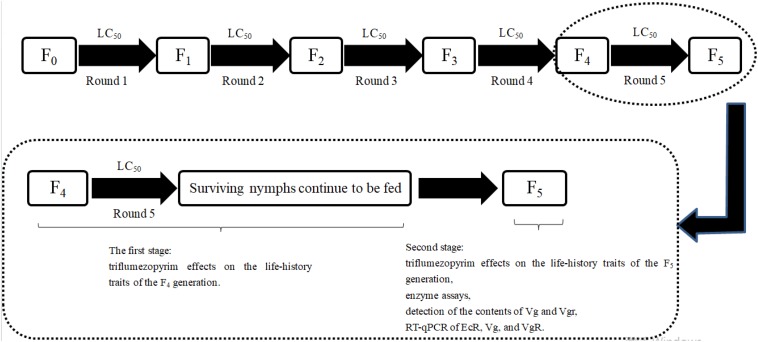
The procedure used to produce the F_5_ generation. According to the bioassay results of the F_0_ generation, 3rd-instar nymphs of susceptible *L. striatellus* were treated with the LC_50_ of triflumezopyrim. Then, the surviving nymphs were collected, and the F_1_ generation was obtained after spawning. The toxicity of triflumezopyrim was assessed in the F_1_ generation, and then the remaining 3rd-instar nymphs of F_1_ were screened with the new LC_50_ value. After five rounds of drug screening, the F_5_ generation was obtained. After treatment of the third-instar F_4_ nymphs, the surviving individuals were selected for observation, as shown in section “Triflumezopyrim Effects on the Life-History Traits of the F_4_ Generation,” and the results are shown in Table 3. Then, a series of experiments to determine sublethal effects and detoxifying enzyme activity were performed on the F_5_ generation. The methods are detailed in sections “Triflumezopyrim Effects on the Life-History Traits of the F_5_ Generation,” “Enzyme Assays,” “Detection of the Contents of Vg and VgR,” and “Reverse Transcriptase-Quantitative Polymerase Chain Reaction of EcR, Vg, and VgR,” and the results are shown in Tables 4–6 and Figures 2–8.

The experimental procedure was performed according to the description of Xiang et al. (2019), with some modifications. The screening operation procedure is shown in Figure 1. According to the bioassay results of the F_0_ generation, the 3rd-instar nymphs of susceptible *L. striatellus* were treated with the LC_50_ of triflumezopyrim. Then, the surviving nymphs were collected, and the F_1_ generation was obtained after spawning. The toxicity of triflumezopyrim was assessed in the F_1_ generation, and then, the survived 3rd-instar F_1_ nymphs were screened with the newer LC_50_ value. After five rounds of drug screening from the F_0_ generation, survival of the F_4_ generation was achieved. Then, 150 surviving nymphs were selected from the F_4_ generation and transferred into flat-bottomed test tubes (diameter × height: 20.0 mm × 145.0 mm) containing fresh rice seedlings. The first 100 nymphs were numbered and used for formal experiments, and the last 50 nymphs were used as substitutes. After emergence, each pair of male and female adults was transferred one by one to a flat-bottomed test tube containing two fresh rice seedlings. If a pair did not mate, they were provided with substitute partners. Third-instar F_0_ nymphs were used for the control treatment. The rice seedlings were replaced every day until the adults died, and the fecundity and longevity of the pair were recorded. The life tables of the F_4_ generation were made at the same time as those of the F_0_ generation.

#### Sublethal Effects of Triflumezopyrim on *L. striatellus*

##### Effects of Triflumezopyrim on the Life-History Traits of the F_4_ Generation

To better understand the effects of triflumezopyrim on the population characteristics of *L. striatellus*, we used an age-stage life table procedure and found that the mean fecundity of the F_4_ generation adults was almost unchanged compared with that of the F_0_ generation adults, and the longevity of the F_4_ generation adults was extended, although the difference was not significant (*P* > 0.05), as shown in Table 3.

##### Effects of Triflumezopyrim on the Life-History Traits of the F_5_ Generation

All developmental times of the F_5_ generation are shown in Table 4. The results indicated that the durations of the egg stage and 1st-instar stage (9.21 and 3.09 days, respectively) in the F_5_ generation were significantly shorter than those in the F_0_ generation (9.67 and 3.38 days, respectively; *P* < 0.05); nevertheless, there was no difference between the two strains during the other nymphal stages (*P* > 0.05).

##### Triflumezopyrim Effects on the Life-History Traits of the F_5_ Generation

The sublethal effects of triflumezopyrim on *L. striatellus* were studied as follows: First, 150 eggs laid on the fifth to sixth day after the emergence and pairing of the sensitive *L. striatellus* and F_4_ insects were collected to serve as the F_0_ (susceptible *L. striatellus* not exposing triflumezopyrim) and F_5_ generations, respectively, and kept in separate test tubes, with 100 for formal experiments and an additional 50 retained as potential replacements. Second, when these eggs became adults, pairs were made according to the method described above. Finally, the population characteristics, including developmental time, longevity, fecundity, and hatching ability, were monitored every day until the pair died. The replaced rice seedlings were dissected under a T-type microscope after 12 days to record the number of unhatched eggs. The life tables of the F_5_ and F_0_ generations were prepared at the same time. The statistical data of the life table were analyzed by using the age-stage, two-sex life table procedure. The life table parameters were analyzed using the program TWOSEX-MSChart (Chi, 2018) and included female age-specific fecundity (*f*_*xj*_), *f*_*x7*_, population age-specific survival rate (*l*_*x*_), population age-specific fecundity (*m*_*x*_), age-stage survival rate (*s*_*xj*_), age-stage–specific reproductive value (*v*_*xj*_), finite rate (λ), net reproductive rate (*R*_0_), intrinsic rate of increase (*r*), mean generation time (*T*), developmental time, adult longevity, adult preoviposition period (APOP), and total preoviposition period (TPOP). The values *f*_*x7*_, *l*_*x*_, *m*_*x*_, *l_*x*_m_*x*_*, *s*_*xj*_, and *v*_*xj*_ were plotted using SigmaPlot 12.3 (Systat Software, Inc, San Jose, California, United States). The mean and standard error of life table parameters were estimated by the bootstrap technique included in TWOSEX-MSChart with 100,000 random resamplings. A paired bootstrap test (TWOSEX-MSChart) program was used to determine the significant differences in population parameters, development duration, and reproductive value of the F_5_ generation compared to the F_0_ generation (TWOSEX-MSChart) (*P* < 0.05).

#### Enzyme Assays

Third-instar nymphs of the F_0_ and F_5_ generations were analyzed for the activity of three detoxifying metabolic enzymes. Carboxylesterase (CarE) activity was determined according to the method described by Asperen (1962). Twenty nymphs (3rd instar) were transferred into a centrifuge tube and stored in liquid nitrogen as quickly as possible, homogenized in 2 mL phosphate buffer (0.04 mol/L phosphate buffer, pH 7.0) ice-bath slurry, and centrifuged at 4°C and 10,000 × *g* for 15 min. Then, the supernatant was transferred into a clean Eppendorf tube as the crude enzyme solution. A mixture of 0.45 mL of phosphate buffer (0.04 mol/L, pH 7.0), 1.8 mL of 3 × 10^–4^ mol/L α-NA solution (containing 3 × 10^–4^ mol/L physostigmine), and 50 μL of diluted enzyme liquid was added to each tube. After thorough mixing, the reaction was performed for 15 min in a constant-temperature bath at 30°C, and then 0.9 mL of staining solution (0.2 g of fast blue-B salt in 20 mL of distilled water plus 50 mL of 5% sodium dodecyl sulfate) was added. The absorbance values were recorded at 600 nm after 5 min in a UV 2000 Spectrophotometer [Unic (Shang Hai) Instruments Incorporated, Shanghai, China].

Glutathione *S*-transferase (GST) activity was determined by using 1-chloro-2,4-dinitrobenzene (CDNB) as a substrate, according to the method of Wang et al. (2018), with minor revisions. Thirty nymphs were placed into a centrifuge tube and stored in liquid nitrogen as quickly as possible. Then, 2 mL of phosphate buffer (0.1 mol/L phosphate buffer containing 1.0 mmol/L EDTA, pH 6.5) was added, and the mixture was centrifuged at 4°C and 10,000 × *g* for 10 min. Then, the supernatant was used as the enzyme solution. The total volume of the reaction system was 2.7 mL, including 2,470 μL of phosphate buffer (0.1 mol/L, pH 6.5), 90 μL of CDNB (15 mmol/L), 50 μL of the enzyme source, and 90 μL of reduced GSH (30 mmol/L). After rapidly shaking the mixture, the absorbance values were monitored within 2 min with a spectrophotometer at 340 nm.

Cytochrome P450 (P450) activity was assayed using the method of Rose et al. (1995) with some modification. One hundred fifty (3rd-instar) nymphs were homogenized on ice with 2 mL of phosphoric acid buffer (0.1 mol/L, pH 7.6, containing 20% glycerol, 0.1 mmol/L EDTA, 0.1 mmol/L DTT, and 0.4 mmol/L phenylmethylsulfonyl fluoride). The homogenate was centrifuged at 4°C and 10,000 × *g* for 10 min to obtain the supernatant, which was stored at low temperature as the enzyme solution. The OD value was determined at 405 nm by adding 100 μL of 4-nitroanisole (2 × 10^–3^ mol/L) and 90 mL of enzyme solution to the enzyme labeling plate, incubating for 3 min at 30°C, and then adding 10 mL of NADPH (9.6 × 10^–3^ mol/L). The OD values were recorded after every 20 s for 2 min, and a standard curve of *p*-nitrophenol was prepared in advance. The specific activity of P450s was finally calculated as nanomoles of *p*-nitrophenol per minute per milligram of protein [nmol/(min mg pro)].

All treatments were performed with three samples (tubes) as biological repetitions, and each enzyme sample was replicated three times as technical repetitions. The total protein content of the enzyme solution was determined by the Bradford method using bovine albumin as a standard (Bradford, 1976). The activities of CarEs, GSTs, and P450s were analyzed using unpaired Student *t*-tests, and the significance level of the results was set at *P* < 0.05.

#### Detection of the Contents of Vg and VgR

Females of the F_0_ and F_5_ generations were collected on the second day after emergence, and triplicates were performed for each treatment. Twenty insects of each sample type were frozen with liquid nitrogen and stored at −80°C. The Vg and VgR contents were detected using the double-antibody sandwich method according to the enzyme-linked immunosorbent assay kit of Alpha Enzyme Biotechnology Co., Ltd. (Shanghai, China). The contents of Vg and VgR were analyzed using unpaired Student *t*-tests, and the significance level of the results was set at *P* < 0.05.

#### Reverse Transcriptase-Quantitative Polymerase Chain Reaction of EcR, Vg, and VgR

Females of the F_0_ and F_5_ generations were collected on the second day after emergence. Total RNA was extracted by using TRIzol reagent (Shanghai Yubo Biological Technology Co. Ltd., Shanghai, China) following the manufacturer’s specifications, and cDNA was synthesized from the total RNA using TransScript cDNA Synthesis SuperMix (Novoprotein Scientific Inc., Shanghai, China) for qPCR. TransStart TopTaq (Novoprotein Scientific Inc.) was used to conduct the reverse transcriptase-quantitative polymerase chain reactions (RT-qPCRs). Primers were designed as shown in Table 1, with the EF-1Fq gene as an internal reference gene (Jia et al., 2015). The RT-qPCRs for each treatment were replicated three times. The relative expressions of EcR (Jia et al., 2015), Vg, and VgR were represented by relative quantification values calculated by the math formula method. The relative expression of the EcR, Vg, and VgR genes was analyzed using unpaired Student *t*-tests, and the significance level of the results was set at *P* < 0.05.

**TABLE 1:** Primers for RT-qPCR.

Gene	Primer	Sequence (5′–3′)	Length
EcR	EcR-F	AAACTGTTGCGCGAGGACCAAATC	187
	EcR-R	AGAACTGCAACAGGTCTTCGACCA	
Vg	Vg-F	GGAAAGCGTGCTTTTGCCAT	149
	Vg-R	ATTGGGGTGGGGAATGCTAC	
VgR	VgR-F	AGACTGTCGCGATGGATCTG	97
	VgR-R	ATGGTTGTCGCATTCTCCCA	
EF-1F	EF-1F-F	CCTTACCCATGTTGGATGCTTATT	95
	EF-1F-R	TGCTTCTGTCTTCCTCTTTCTTCC	

### Results

#### Toxicity of Triflumezopyrim on 3rd-Instar Nymphs of *L. striatellus*

To understand the changes in the susceptibility of *L. striatellus* under the screening pressure of triflumezopyrim, we used the rice seedling dipping method to evaluate the toxicity of triflumezopyrim and found that the LC_50_ values of triflumezopyrim on 3rd-instar nymphs of the F_0_ and F_4_ generations were 0.443 ± 0.171 μg/mL and 5.682 ± 1.498 μg/mL, respectively, with a resistance ratio of 12.84-fold (Table 2).

**TABLE 2:** The toxicity of triflumezopyrim on the 3rd-instar nymphs of the F_0_ and F_4_ generations of *L. striatellus*.

Strain	No. of tested insects	Slope ± SE	LC_50_ (μ g/mL), 95% CL	χ ^2^ (*df*)	*Pr*
F_0_	270	1.202 ± 0.349	0.443 (0.272–0.619)	4.240 (13)	0.8733
F_4_	270	1.736 ± 0.242	5.682 (4.184–7.271)	2.790 (13)	0.8729

*The results are presented as the number of tested insects, standard error, 95% confidence limits, *χ*^2^ value, degrees of freedom (*df*), and *Pr* calculated by SAS 9.2.*

**TABLE 3:** The adult longevity and fecundity of the F_0_ and F_4_ generations of *L. striatellus*.

Parameter	F_0_	F_4_	*P*
Longevity (d) ± SE	20.12 ± 0.92 a	21.58 ± 1.52 a	0.416 > 0.05
Mean fecundity (eggs/female) ± SE	213.16 ± 16.30 a	209.39 ± 22.90 a	0.895 > 0.05

*The results are presented as the adult longevity and fecundity of the F_0_ and F_4_ generations. The rows represent the average (±SE). Different lowercase letters indicate significant differences according to Duncan multiple-range test (*P* < 0.05).*

**TABLE 4:** The developmental duration of the egg, nymph, and adult stages in the F_0_ and F_5_ generations of *L. striatellus*.

Developmental stage	Strain		*P*
	F_0_	F_5_	
Egg	9.67 ± 0.05 a (100)	9.21 ± 0.13 b (100)	0.0008 < 0.05
1st instar nymph (d)	3.38 ± 0.10 a (93)	3.09 ± 0.07 b (89)	0.0146 < 0.05
2nd instar nymph (d)	1.92 ± 0.06 a (92)	1.84 ± 0.05 a (89)	0.2958 > 0.05
3rd instar nymph (d)	2.25 ± 0.07 a (92)	2.44 ± 0.08 a (89)	0.0628 > 0.05
4th instar nymph (d)	2.88 ± 0.07 a (92)	3.07 ± 0.09 a (89)	0.0904 > 0.05
5th instar nymph (d)	4.08 ± 0.10 a (87)	4.15 ± 0.13 a (87)	0.6826 > 0.05
Adult (d)	20.78 ± 0.97 a (87)	18.97 ± 0.80 a (87)	0.1386 > 0.05
Preadult (d)	24.09 ± 0.13 a (87)	23.77 ± 0.13 a (87)	0.0775 > 0.05

*The results are presented as the developmental duration of the egg, nymph, and adult stages in the F_0_ and F_5_ generations of *L. striatellus*. The rows represent the average (±SE) and degrees of freedom (*df*). Different lowercase letters indicate significant differences according to Duncan’s multiple-range test (*P* < 0.05). The numbers in parentheses represent the numbers of test insects at particular stages.*

The fecundity, APOP, TPOP, and longevity of the F_5_ generation are displayed in Table 5. The mean fecundity of the F_5_ generation (141 eggs) was significantly lower than that of the F_0_ generation (186 eggs) (*P* < 0.05), whereas the APOP, TPOP, and adult longevity were not significantly different between the two strains (*P* > 0.05). The proportions of males to females in the F_5_ and F_0_ generations were 1:0.85 and 0.89:1, respectively.

**TABLE 5:** The adult longevity and female fecundity of the F_0_ and F_5_ generations of *L. striatellus*.

Parameters	Gender	Strain		*P*
		F_0_	F_5_	
Adult longevity (d)	Male	19.18 ± 1.22 a (40)	18.11 ± 1.14 a (46)	0.5143 > 0.05
	Female	22.15 ± 1.45 a (47)	19.93 ± 1.11 a (41)	0.2104 > 0.05
Adult preoviposition period (d)	Female	1.87 ± 0.11 a (47)	1.7 ± 0.13 a (41)	0.3436 > 0.05
Total preoviposition period (d)	Female	26.11 ± 0.18 a (47)	26.15 ± 0.18 a (41)	0.883 > 0.05
Mean fecundity (eggs/female)	Female	186.57 ± 14.73 a (47)	141.15 ± 12.34 b (41)	0.0156 < 0.05

*The results are presented as the adult longevity and female fecundity of the F_0_ and F_5_ generations of *L. striatellus*. The rows represent the average (±SE) and degrees of freedom (*df*). Different lowercase letters indicate significant differences according to Duncan multiple-range test (*P* < 0.05). The numbers in parentheses represent the numbers of test insects at particular stages.*

The sublethal effects of triflumezopyrim on population dynamics were calculated with a bootstrapping procedure based on a life table (Table 6). The intrinsic rate of increase (*r*), finite rate of increase (λ), and net reproductive rate in the F_5_ generation (0.12, 1.13, and 57.87, respectively) were all lower than those in the F_0_ generation (0.14, 1.15, and 87.69, respectively) (*P* < 0.05). However, there was no difference in the average generation time (*T*) between these two strains (*P* > 0.05).

**FIGURE 2:**
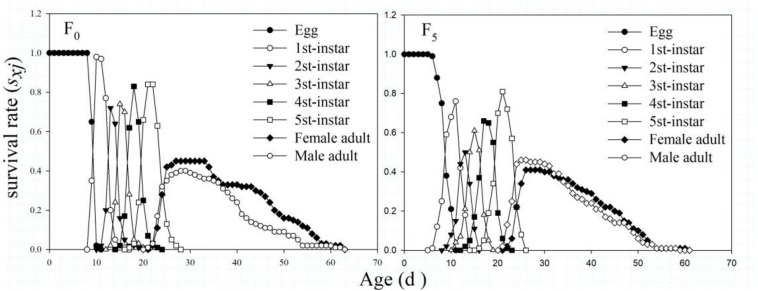
The age-stage-specific survival rate (*s*_*xj*_) of the F_0_ and F_5_ generations of *L. striatellus*.

The *s*_*xj*_ values in the F_0_ and F_5_ generations indicated that their growth curves had substantial overlap and complex growth relationships among individuals. The number of nymphs that could complete development in the two strains was not significantly different (83 and 77%, respectively). In addition, the specific age survival rate of male adults in the F_0_ generation was higher than that of female adults, whereas a different pattern was observed in the F_5_ generation, and the *s*_*xj*_ values of male adults were similar to those of female adults (Figure 2).

**TABLE 6:** The population parameters of the F_0_ and F_5_ generations of *L. striatellus*.

Strain	Intrinsic rate of increase (d^–1^), *r*	Finite rate of increase (d^–1^), λ	Net reproductive rate, *R*_0_	Mean generation time (d), *T*
F_0_	0.14 ± 0.004 a	1.15 ± 0.005 a	87.69 ± 11.615 a	32.69 ± 0.414 a
F_5_	0.12 ± 0.004 b	1.13 ± 0.005 b	57.87 ± 8.464 b	32.8 ± 0.399 a
*P*	0.0269 < 0.05	0.0267 < 0.05	0.0373 < 0.05	0.7842 > 0.05

*The results are presented as the intrinsic rate of increase (*r*), finite rate of increase (λ), net reproductive rate (*R*_0_), and mean generation time (*T*) of the F_0_ and F_5_ generations of *L. striatellus*. The rows represent the average (±SE). Different lowercase letters indicate significant differences according to Duncan multiple-range test (*P* < 0.05).*

**FIGURE 3:**
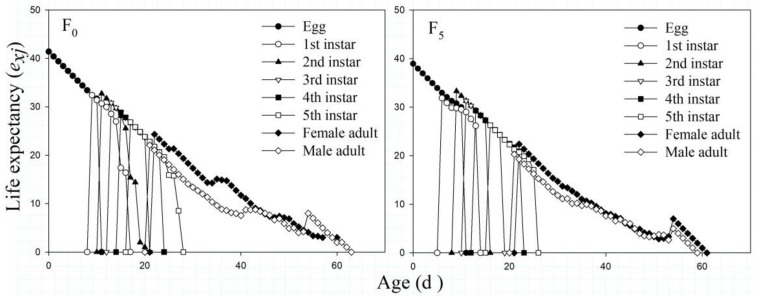
The age-stage-specific life expectancy (*e*_*xj*_) of the F_0_ and F_5_ generations of *L. striatellus.*

According to Figure 3, the *e*_*xj*_ values of the F_0_ and F_5_ generations generally decreased with age *x* and phase *j*. For the F_0_ and F_5_ generations, the highest *e*_*xj*_ values all appeared at the egg stage, at 41.41 and 38.95 days, respectively. Overall, the *e*_*xj*_ values of the F_5_ generation were lower than those of the F_0_ generation, whereas the *e*_*xj*_ values of the female adults were higher than those of the male adults. Among all the adults, the *e*_*xj*_ values of females in the F_0_ generation were the highest.

**FIGURE 4:**
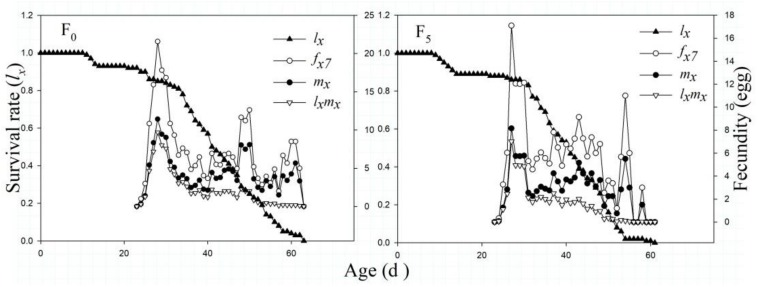
The age-specific survival rate (*l*_*x*_), female age-specific fecundity (*f*_*x7*_), age-specific fecundity of the total population (*m*_*x*_), and age-specific net maternity (*l_*x*_m_*x*_*) of the F_0_ and F_5_ generations of *L. striatellus*.

The *l*_*x*_ results indicated that the *l*_*x*_ curves of the F_0_ and F_5_ generations generally first increased and then decreased. The highest peak fertility in the F_0_ and F_5_ generations was on the 28th and 27th days, with the highest average yield of 11.0 and 17.1 hatched eggs, respectively (*P* < 0.05). The female *f*_*x*_ curve of the F_5_ generation suggested a lower and less variable value than that of the F_0_ generation. Similarly, the curves of *m*_*x*_ and *l_*x*_m_*x*_* showed a declining trend in the F_5_ generation (Figure 4).

**FIGURE 5:**
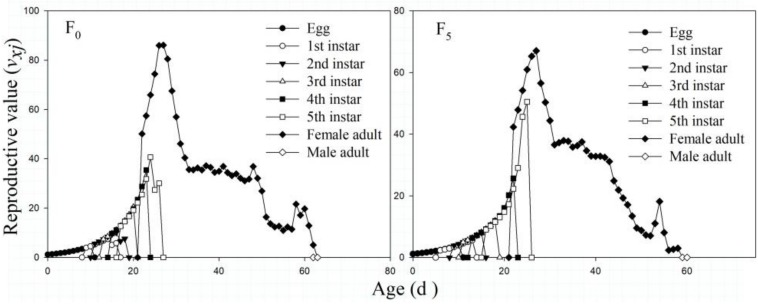
The age-stage–specific reproductive value (*v*_*xj*_) of the F_0_ and F_5_ generations of *L. striatellus.*

The *v*_*xj*_ values in the F_5_ generation were lower than those in the F_0_ generation for the female adult stage and total *v*_*xj*_. The *v*_*xj*_ values of all strains gradually increased from the egg stage, and that in the F_0_ generation reached its maximum (124.65 eggs) on the 23rd day, especially for female adult reproduction, which showed a peak value of 86.11 eggs on the 27th day. However, the *v*_*xj*_ values in the F_5_ generation reached their highest peaks (111.45 eggs) on the 25th day, and those of female adults reached their maximum (67.06 eggs) on the 27th day (Figure 5).

### Enzymatic Activity in the F_0_ and F_5_ Generations

**FIGURE 6:**
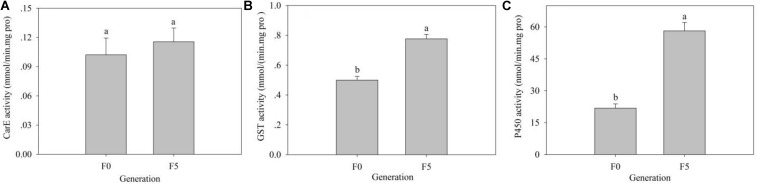
The effects of triflumezopyrim on the activity of the detoxification enzymes CarE **(A)**, GST **(B)**, and P450 **(C)** in 3rd-instar nymphs of *L. striatellus*. The activities of CarEs, GSTs, and P450s in 3rd-instar nymphs of *L. striatellus* are presented as the mean of three replications ± SE. Means followed by the same letters did not differ significantly (*P* > 0.05) according to the analysis of variance test. The F_1,4_ values of different treatments of CarEs, GSTs, and P450s in 3rd-instar nymphs of *L. striatellus* were 0.367, 50.654, and 68.502, and the *P* values of CarE, GST, and P450 in 3rd-instar nymphs of *L. striatellus* were 0.577 > 0.05, 0.002 < 0.05, and 0.001 < 0.05, respectively.

Insect detoxifying metabolic enzymes could be induced by xenobiotics, including insecticides. We assayed the activity of three main detoxifying metabolic enzymes, including CarEs, GSTs, and P450s, in the F_0_ and F_5_ generations of *L. striatellus*. Compared with the F_0_ generation, the activities of the three detoxifying metabolic enzymes of the F_5_ generation were increased to varying degrees. The GST [0.78 mmol/(min mg pro)] and P450 activities [58.15 nmol/(min mg pro)] were significantly higher in the F_5_ generation than in the F_0_ generation [0.50 mmol/(min mg pro) and 21.84 nmol/(min mg pro), respectively] (*P* < 0.05), whereas the CarE activity was not significantly different between the F_0_ and F_5_ generations (*P* > 0.05) (Figure 6).

### Contents of Vg and VgR in the F_0_ and F_5_ Generations

**FIGURE 7:**
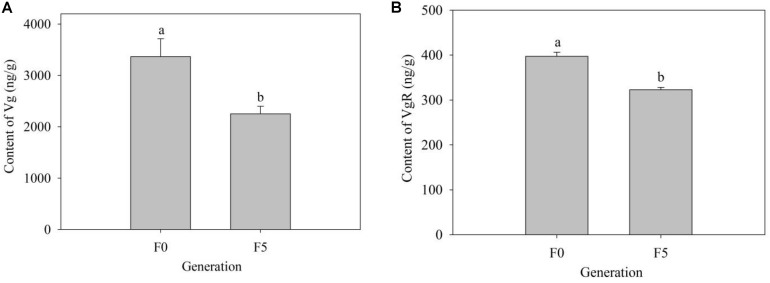
**(A,B)** Contents of Vg and VgR in female adults of the F_0_ and F_5_ generations of *L. striatellus*. The bars represent the average (±SE). The contents of Vg and VgR are presented as the mean of three replications ± SE. The different lowercase letters above the columns indicate significant differences at the *P* < 0.05 level according to the analysis of variance test. The F_1,4_ values of Vg and VgR contents in the female adults of the F_0_ and F_5_ generations were 8.786 and 49.943, respectively, and the *P* values of Vg and VgR contents in the female adults of the F_0_ and F_5_ generations were 0.041 < 0.05 and 0.002 < 0.01, respectively.

To further explore the mechanism of triflumezopyrim on the growth and development of *L. striatellus*, we detected the contents of Vg and VgR in the F_0_ and F_5_ generations. Compared with those in the F_0_ generation (2,251.80 and 148.33 ng/g), the contents of Vg and VgR in the F_5_ generation (3,367.35 and 397.35 ng/g) both increased significantly (*P* < 0.05) (Figure 7).

### Relative Expression of EcR, Vg, and VgR

**FIGURE 8:**
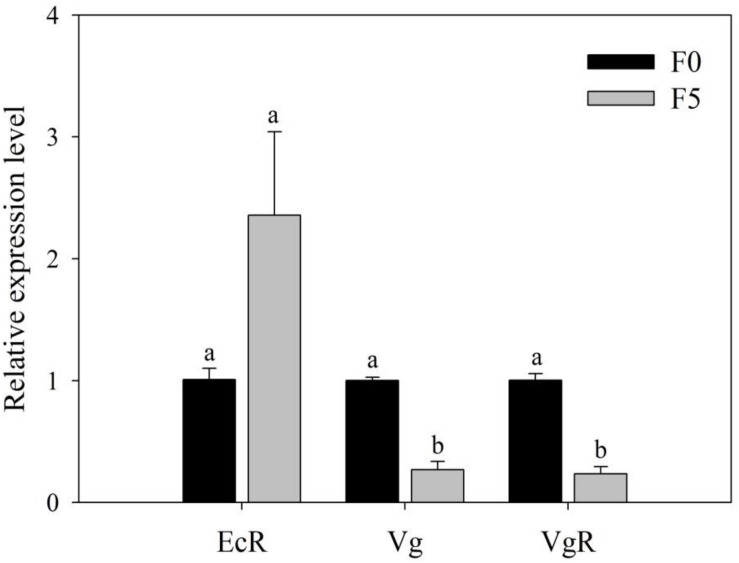
Relative expression level of EcR, Vg, and VgR genes in female adults of the F_0_ and F_5_ generations of *L. striatellus*. The bars represent the SE of three replications. Different lowercase letters indicate significant differences according to Duncan multiple-range test (*P* < 0.05). The F_1,4_ values of the relative expression levels of EcR, Vg, and VgR genes in the female adults of the F_0_ and F_5_ generations were 3.792, 726.682, and 49.943, respectively, and the *P* values of the relative expression of the EcR, Vg, and VgR genes in female adults of the F_0_ and F_5_ generations were 0.577 > 0.05, 0.041 < 0.05, and 0.002 < 0.01, respectively.

To seek direct evidence of triflumezopyrim having affected the contents of Vg and VgR of *L. striatellus*, we detected the relative expression of EcR, Vg, and VgR in *L. striatellus* and found that the relative expression of Vg and VgR (0.27- and 0.23-fold, respectively) in female adults of the F_5_ generation was significantly decreased compared with that in the F_0_ generation (*P* < 0.05); the relative expression of EcR (2.36-fold) was slightly increased, but no significant difference was found between the two treatments (*P* > 0.05; Figure 8).

## Discussion

Research on insect life tables is one of the imperative aspects of insect population dynamics, and changes in insect populations are influenced by multiple factors, including food, temperature, light, their respective hosts (Tuan et al., 2014; Zhang et al., 2015; Qin et al., 2017), and especially the different kinds of chemical pesticides. Our results showed that the fecundity (including *r*, λ, and *R*_0_) of *L. striatellus* was significantly decreased following triflumezopyrim treatment and population expansion of *L. striatellus* was suppressed. This was consistent with an earlier report by Liao et al. (2019), who declared that the sublethal effects of sulfoxaflor reduced the survival and reproductive capability of *Nilaparvata lugens* in successive generations. Xiang et al. (2019) also found that the number of *Sogatella furcifera* (Hemiptera: Delphacidae) eggs decreased with the LC_25_ of sulfoxaflor. In addition, we found that the developmental stage of the F_5_ generation was not greatly affected, and the values of *T*, APOP, and TPOP did not change significantly, which was inconsistent with the findings of some previous reports. For instance, low sublethal concentrations of sulfoxaflor could stimulate the fertility of *S. furcifera*, and the APOP, TPOP, and *T* values were significantly prolonged, even though the longevity, fecundity, and egg-hatching ability of *N. lugens* were not significantly affected by the LC_30_ of triflumezopyrim (Xu et al., 2019). We speculated that different insects respond differently to insecticide stress.

Insects will develop resistance when successively exposed to the same insecticide. Kwon et al. (2019) reported that *L. striatellus* was reared for nine generations with carbofuran selection, and its resistance was increased 14-fold. The development of resistance is closely related to changes in three major detoxification metabolic enzymes in insects, including CarEs, GSTs, and P450s. According to the results of Asaduzzaman et al. (2019), the overexpression of P450 genes in *L. striatellus* has resulted in deltamethrin resistance. Abdalla Elzaki et al. (2016) also reported that imidacloprid resistance in *L. striatellus* was related to increased P450 activity. Meanwhile, there have also been many reports that enhanced GST activity has contributed to the development of resistance in insects, similar to the study of Meng et al. (2019a), who alleged that GSTs played an essential role in the detoxification of malathion in *Bactrocera dorsalis*. In our research, P450 and GST activities were significantly increased in the F_5_ generation, and we speculated that the increase in P450 and GST activities could collaboratively enhance the triflumezopyrim resistance of *L. striatellus*. Whether the susceptibility and detoxification enzyme activities of the F_5_ generation would return to their previous levels in the F_0_ generation when the pressure of triflumezopyrim was removed should be further studied, although some reports have declared that resistance levels decreased when insects with high or moderate resistance levels were exposed to no insecticides (Ishtiaq et al., 2014; Abbas et al., 2015).

We found that the reproductive capacity of the F_5_ generation decreased significantly. It has been reported that the development of drug resistance is often accompanied by a number of adverse factors, such as shortened total life span and reduced fertility (Zhang et al., 2018). To further understand its mechanism, we measured the expression levels of three genes closely related to development and reproduction. Vitellogenin is a vital reproduction-related protein that is traditionally considered to be an adequate parameter for evaluating female fertility in insects (Li et al., 2010; Zhao et al., 2018). For example, the decreased expression of Vg was found to have negative impacts on the fecundity of *Chilo suppressalis* and *Apolygus lucorum* (Huang et al., 2016; Zhen et al., 2018). Vitellogenin receptor is a necessary receptor for Vg to function (Schneider, 1996; Snigirevskaya et al., 1997). Notably, a decrease in VgR expression can inhibit the function of Vg. In our experiment, the mRNA expression levels of Vg and VgR in F_5_ generation female adults were obviously lower than those in the F_0_ generation. Combined with the fact that the fecundity of the F_5_ generation was significantly reduced, these data suggested that reduced Vg mRNA expression in the triflumezopyrim-resistant strain might significantly influence the fecundity of *L. striatellus*. Furthermore, we found that the mRNA expression level of EcR in F_5_ generation female adults showed a slight increase compared with that in the F_0_ generation, even though no significant differences were observed. This finding suggested that triflumezopyrim may modulate the molting hormone pathway in *L. striatellus*, and this result could explain the decreased expression of Vg and the shortened duration of the egg, nymph, and adult stages in the F_5_ generation to some extent. Moreover, we found that the Vg and VgR contents in the F_5_ generation were also significantly decreased; these results directly confirm the changing trend of Vg and VgR gene expression.

## Conclusion

In this study, an age-stage life table procedure was used to evaluate the effects of a sublethal concentration (LC_50_) of triflumezopyrim on the biological parameters of *L. striatellus* for the first time. The detoxification enzyme activities of GSTs and P450s were induced by triflumezopyrim in the F_5_ generation compared to those in the F_0_ generation. The contents and relative expression of Vg and VgR in the F_5_ generation were also significantly decreased compared to those in the F_0_ generation.

## Data Availability Statement

All datasets generated for this study are included in the article/supplementary material.

## Author Contributions

SZ, FG, CG, YZ, and XW conceived the study. SZ, FG, LC, and LS conducted the experiments. SZ drafted the preliminary manuscript and interpreted the results. XW, SZ, CJ, and AH refined and approved the final manuscript.

## Conflict of Interest

The authors declare that the research was conducted in the absence of any commercial or financial relationships that could be construed as a potential conflict of interest.
